# Effects of fibre-rich rye milling fraction on the functional properties and nutritional quality of wholemeal rye bread

**DOI:** 10.1007/s13197-019-04050-8

**Published:** 2019-08-26

**Authors:** Piotr Kołodziejczyk, Jan Michniewicz, Maciej S. Buchowski, Hanna Paschke

**Affiliations:** 1 Institute of Food Technology of Plant Origin, Poznan University of Life Sciences, Wojska Polskiego 31, 60-624 Poznan, Poland; 2 Division of Gastroenterology, Department of Medicine, Vanderbilt University Medical Center, 1161 21st Avenue South, Nashville, TN 37321 USA

**Keywords:** Wholemeal rye bread, High-fibre rye flour, Bread supplementation, Nutritional quality of bread

## Abstract

The goal was to assess the effects of partial replacement of wholemeal rye flour with 30%, 40% and 50% of the high-fibre rye flour (HFRF) on nutritional quality and sensory and physicochemical characteristics of breads. The HFRF supplemented breads (SB30, SB40, SB50) were compared in their nutrients and energy contents, physicochemical and sensory properties, and in vitro digestibility to the control bread (CB). There were no significant differences in shape and volume of loaves, crusts and crumbs appearance, taste and smell of two supplemented breads (SB30 and SB40) and the CB. Compared to the CB, all supplemented breads contained significantly more soluble and insoluble fibre, arabinoxylan and β-glucan, but less available saccharides, including rapidly available glucose. Bread with 40% HFRF (SB40) yielded both, improved nutritional quality and acceptable sensory characteristics comparable to the CB. An in vitro overall digestibility of the SB40 was lower than that of the CB but the losses of dietary fibre and its components after enzymatic digestion were comparable between both breads. In conclusion, rye bread supplemented with 40% of the HFRF had improved nutritional quality and acceptable sensory and physicochemical characteristics and could be considered as an option to commonly consumed wholemeal rye bread.

## Introduction

The important role cereals and particularly cereal dietary fibre (DF) in human nutrition, enhanced health and lifestyle-related non-communicable diseases prevention has been documented in a large number of laboratory, clinical and epidemiological studies (Slavin 2003; Ye et al. 2012). In many Western countries, the typical diet contains an unacceptably low level of DF (< 20 g per day), which is 20% less than the minimum recommended for adults (25 g/day) by the European Food Safety Authority (EFSA 2010).

Thus, bread is an attractive vehicle for delivering nutritionally beneficial fibre and other bioactive compounds because it is widely consumed and available in a range of types at affordable prices. Potential approaches to increasing the delivery of compounds with high nutritional value in breads include increasing the use of “high extraction” and wholemeal flours. Among flours used in bread making, rye flour is considered one of the best raw materials to produce bread with a significantly higher DF content than whole-grain wheat flour or any other flours made from non-bread cereals (Boskov Hansen et al. 2002; Rakha et al. 2010). Moreover, consumers in Northern and Eastern Europe, including Poland, prefer wholemeal rye soft bread for its excellent and appetizing aroma and taste. For example, in Finland, this type of bread is considered the best source of DF (Poutanen et al. 2014). Therefore, there is a need to develop new types of rye bread that combine improved nutritional quality and health benefits with attractive sensory characteristics. Based on our knowledge and experience, we expect that this task could be fulfilled by wholemeal rye bread enriched with high-fibre rye flour containing (compared to wheat flour) higher amounts of DF, especially its soluble fraction. In the absence of scientific information in this field, in the literature review we used the information presented in numerous different studies on the impact of wheat DF on wheat bread.

Most recent research concerning this has focused on the effects of adding different DF sources on quality, functional and nutritional properties of wheat bread (Symonds and Brennan 2004; Peressini and Sensidoni 2009; Angioloni and Collar 2011; Raggaee et al. 2011; Rizzello et al. 2012; Almeida et al. 2013; Koletta et al. 2014). Several studies demonstrated the usefulness of various whole-grain cereals, different types of bran and milling by-products as concentrated sources of DF (Symonds and Brennan 2004; Raggaee et al. 2011; Rizzello et al. 2012; Almeida et al. 2013; Nordlund et al. 2013; Koletta et al. 2014). The applicability of some single DF components such as β-glucan, inulin, different type of resistance starches, glucooligosaccharides, cellulose, guar and locust bean gum, xanthan has also been examined (Symonds and Brennan 2004; Peressini and Sensidoni 2009; Angioloni and Collar 2011; Raggaee et al. 2011; Almeida et al. 2013). Most of these studies were conducted to find out the maximum replacement level of white wheat flour with a high-fibre source at which consumer-acceptable bread can be produced. Majority of these reports indicates that the incorporation of various DF sources in bread often leads to changes in loaf volume, crumb texture, colour, taste and other properties, thus reducing acceptability by consumers. Benefits of DF addition in bread are improved nutritional quality such as reduced contents of readily available glucose and energy and/or increased content of some vitamins, minerals and phytochemicals (Tas and El 2000; Angioloni and Collar 2011; Raggaee et al. 2011; Rizzello et al. 2012; Nordlund et al. 2013). The extent of these benefits depends on the level of substitution, type of DF source, composition and molecular characteristics of DF, type, and quality of bread flour. In the literature, there are many examples of chemical, mechanical, thermal, enzymatic and microbial modifications of DF cereal-based products designed to minimise adverse effects and maximise nutritional benefits of DF in wheat bread (Rizzello et al. 2012; Nordlund et al. 2013). These treatments of DF cereal-based products have been primarily used to improve their technological functionality with subsequent improvements in bread quality as well as to enhance nutritional quality. Several small human feeding studies have investigated the biological effects of DF-enriched breads. For example, a few studies examined the effect of wholemeal rye bread on blood cholesterol, postprandial glucose metabolism and insulin secretion, and bowel function (McIntosh et al. 2003; Lappi et al. 2014). The conclusion from these and several epidemiological studies was that increased consumption of breads and other cereal products with high nutritional quality could contribute to improved health outcomes for consumers.

Therefore, the goal of this study was to assess the effects of the wholemeal rye flour (WRF) supplementation with the high-fibre rye flour (HFRF) on nutritional quality, and sensory and physicochemical characteristics of bread. The criteria for assessing nutritional quality of bread were contents of minerals, protein, total and soluble and insoluble fractions of dietary fibre, arabinoxylan, β-glucan, fructan, available saccharides including rapidly available glucose, and energy content. We hypothesised that compared to control bread, wholemeal rye breads supplemented with 30%, 40% or 50% of HFRF will have improved the nutritional quality without significant change in their sensory and physicochemical characteristics.

## Materials and methods

### Raw materials

The commercial wholemeal rye flour type 2000 and grain of rye hybrid variety *Visello* obtained from Kleinwanzlebener Saatzucht Polska Sp. Z o.o., (Kondratowice, Poland) harvested in 2011 were used.

### Preparation of the high-fibre rye flour

**Fig. 1:**
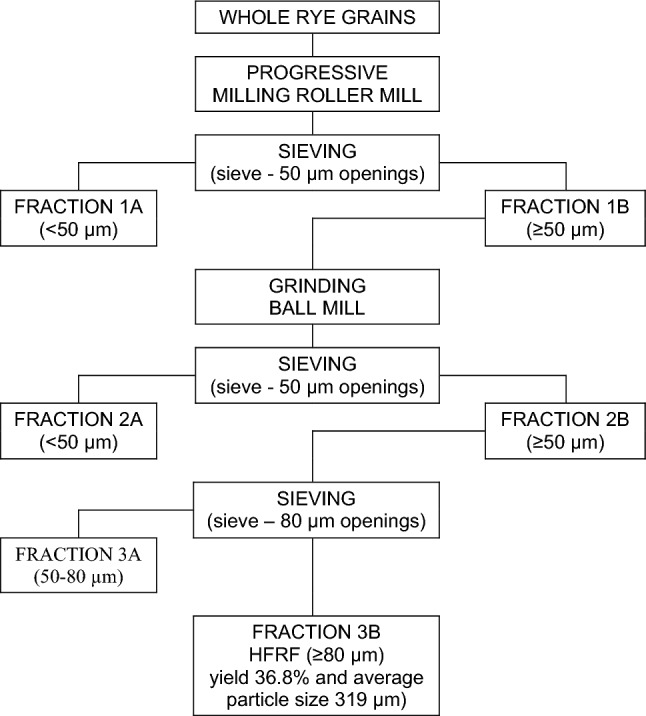
Rye grain dry fractionation for the preparation of high-fibre rye flour (HFRF)

The schematic process of rye milling is given in Fig. 1 (Kolodziejczyk et al. 2018). Cleaned grains tempered to 14.5% of kernel moisture were milled using a laboratory roller mill (Quadrumat Senior, Brabender GmbH, Germany) at the default settings. The obtained wholemeal flour was passed through break rollers and then twice through the mill reduction rollers without sieving. The received fine wholemeal flour was separated in a sieve shaker (AS 200 Basic, Retsch GmbH, Germany) on a sieve with 50 μm openings into a coarse fraction (1B ≥ 50 μm) and an endosperm-rich fraction (1A < 50 μm) for 20 min. The fraction 1A was discarded. The fraction 1B was reground in a laboratory porcelain ball mill type-6 (Machine-Building Factory, Lodz, Poland) for 180 min. The internal bowl depth was 22.5 cm. The diameters of ball and bowl were 2.4 cm and 22.5 cm, respectively. The regrounded fraction 1B was sieved on a sieve with 50 μm openings into fractions 2A (< 50 μm) and 2B (≥ 50 μm). The fraction 2A was discarded, and the fraction 2B was separated on sieves with 80 μm openings into fractions 3A (< 80 μm) and 3B (≥ 80 μm). The fraction 3A was discarded, while the fraction 3B (36.8% of the initial grain weight), with the average particle size 319 μm, was the final high-fibre rye flour (HFRF) product.

### Bread making procedure

Wholemeal rye flour blends were prepared by supplementing the commercial wholemeal rye flour type 2000 (WRF) with 30%, 40% and 50% of HFRF. We have chosen the 30, 40 and 50% of supplementation with HFRF based on pilot studies from our laboratory. The control bread (CB) and supplemented breads (SB30, SB40, and SB50) were prepared in a two-step sourdough making procedure. In the first step, a sour was prepared by mixing 160 g of the WRF, 10 g of the starter and 80 ml of water. The sour was fermented for 20 h at room temperature (~ 20 °C, ~ 60% RH). In the second step, the sour (240 g) was mixed with the WRF (240 g) and water (240 ml). The obtained sourdough was fermented for 3 h at 30 °C and 90% RH in a proofing chamber (Basic Model Jeio Tech, Jeio Tech Co., Ltd., South Korea). The sourdough (720 g), the WRF or its blend with the HFRF (600 g), water (430 ml), salt (15 g) and fresh yeast (10 g) were mixed for 5 min in a mixer (KitchenAid Aristan 5, KitchenAid, USA). The dough was proofed (30 min, 30 °C, 90% RH) and divided into 4 equal pieces (400 g), hand-moulded into loaves and proofed in baking pans (34 °C, 90% RH) for 30 min. Breads were baked in a laboratory electric convection oven (Gierre, IK Interklimat S.p.A., Italy) at 220 °C for 25 min. After baking, bread loaves were weighted and cooled to room temperature (~ 20 °C). After 1 h of cooling, the breads’ volume was measured and the colour assessed. The sensory evaluation of breads was carried-out 20 h after baking. The breads were freeze-dried, ground, sieved (500 μm), and stored at 4 °C in airtight glass containers for further testing. Four bread batches for each recipe were baked.

### Analytical methods

The chemical compounds were determined according to the AACC Approved Methods and ICC Standard (AACC Approved Methods 2000; ICC-Standards 1998). The moisture content of flours was determined according to ICC-Standard 110. The breads moisture was determined by oven drying (Model ED53 Binder, Binder GmbH, Germany) for 12 h at 105 °C. The flours and breads components were calculated per 100 g of dry matter and 100 g as eaten, respectively.

### Macronutrients content of flours and breads

The ash content was determined according to ICC-Standard 104. The total nitrogen and lipids contents were determined according to the AACC Approved Methods (46-10 and 30-10, respectively). The protein content was calculated using a nitrogen conversion factor for rye (6.25). The total starch in both flours was analysed according to the AACC Approved Methods 76-13 using the Megazyme total starch assay kit K-TSTA (Megazyme International Ireland Ltd., Wicklow, Ireland). The content of the total reducing saccharides was determined colorimetrically with 3,5-dinitrosalicylic acid (James 1999).

### Energy content of bread

The energy content was calculated as a sum of the energy of major chemical constituents contained in 100 g of bread as eaten (kcal/100 g and kJ/100 g). It was assumed that the energy value of 1 g of protein, available starch, and free sugars was 4 kcal (17.2 kJ), 1 g of fat was 9 kcal (38.9 kJ) and 1 g of total dietary fibre and fructan was 2 kcal (8 kJ) (FAO 2003; Maziarz 2013).

### Dietary fibre and its components

The soluble DF (SDF) and insoluble DF (IDF) contents were determined according to AACC Method 32-07 using the Megazyme total dietary fibre assay kit K-TDFR-200A. The total DF (TDF) was calculated as a sum of SDF and IDF. The major DF components were determined as total arabinoxylan and water-soluble arabinoxylan (WE) according to the colorimetric method of Hashimoto et al. (1987). The total β-glucan, water-insoluble β-glucan (WUE) and fructan were estimated by the AACC Approved Methods 32-23 and 32-32, respectively. The water-insoluble arabinoxylan (WUE) was calculated as a difference between total arabinoxylan and WE arabinoxylan. The water-soluble β-glucan (WE) was the difference between total β-glucan content and its WUE fraction. The β-glucan and fructan contents were analysed using the Megazyme β-glucan mixed linkage and fructan assay kits K-BGLU and K-FRUC, respectively. All analyses were run in triplicates.

## Physicochemical characteristics of breads

### Loaf volume and specific volume

The loaf volume (cm^3^) was determined by the rapeseed displacement method 1 h after baking according to AACC Method 10-05. The specific volume was calculated by dividing the bread volume by its weight (cm^3^/g). Data are mean of three measurements performed on three loaves from different batches.

### Acidity

Total titratable acidity expressed as acidity degrees (°) was measured according to the Standard Methods of the Arbeitsgemeinschaft Getreideforschung (Boskov Hansen et al. 2002).

### Bread colour

The colour of crust and crumb was assessed using Chroma Meter CR 410 (Konica Minolta, Japan) in the CIELAB System (L*, a* and b*). Results were expressed as the total colour difference (∆E*) between the CB and the supplemented breads and the browning index (BI). The ∆E* and BI values were calculated according to the following equations (Peressini and Sensidoni 2009; Angioloni and Collar 2011):

$$ \begin{aligned} \Delta E^{*} & = \sqrt {\Delta L^{*2} + \Delta a^{*2} + \Delta b^{*2} } \\ {\text{BI}} & = 100{-}{\text{L}} \\ \end{aligned} $$

where ∆L*—lightness difference, ∆a*—redness difference and ∆b*—yellowness difference, L—lightness.

Values of ∆E* and BI are means of 10 measurements performed on three loaves from different batches.

### Sensory evaluation

The breads were evaluated by 6 experienced and trained panellists (2 males and 4 females, mean age: 32 years, range: 23–60 years) using a 20-point rye bread scoring system according to the Standard Methods of the Arbeitsgemeinschaft Getreideforschung (Drews and Seibel 1976). The sensory scoring system and its features were discussed with the panellist during three training sessions. The assessing system is comprised of four factors (shape and volume, crust, crumb and sensory test) and bread acidity. Each factor was assessed individually and given a score. The minimum score was 0 and maximum scores for shape, volume and crust were 2 points, crumb 10 points (grain 5, elasticity 3 and texture 2), and sensory test 5 points. Acidity was scored either 0 or 1 point. One point was assigned for typical acidity values of rye wholemeal bread (8°–14°). The total score was the sum of individual scores. Based on the 20-point scoring system, bread was characterized as excellent (19–20 points), good (17–18 points), satisfactory (16 points) and unsatisfactory (< 16 points).

### In vitro starch digestibility

Total starch, rapidly digestible starch, slowly digestible starch and resistant starch, rapidly available glucose and free glucose were determined using the method of Englyst et al. (1992). Glucose content after enzymatic starch hydrolysis was determined using the Megazyme glucose oxidase assay kit K-GLUC. The Starch Digestion Index (SDI) was calculated as a ratio of rapidly digestible starch and total starch (%). Data are reported as the mean of three measurements of loaves from three experiments.

### In vitro enzymatic digestion

The CB and SB40 were digested enzymatically using the method developed by Aura et al. (1999) that applies 24-h dialysis treatment (Sigma D9527, cut-off 12 400 Da) with 6 times change of deionized water. The enzymatic digestion was performed in the following stages: (a) salivary α-amylase (50 U/1.5 g of sample) treatment at pH 6.5 and 37 °C for 5 min; (b) pepsin (2 mg/mL of 20 mM hydrochloric acid per sample) treatment at pH 2.5 and 37 °C for 2 h; (c) bovine bile (600 mg/4 mL of 150 mM sodium bicarbonate per sample), pancreatin (75 mg/4 mL of 150 mM sodium bicarbonate per sample) and mucin (75 mg/mL of distilled water per sample) treatment at pH 6.5–7.5 and 37 °C for 3 h; (d) dialysis at 4 °C for 24 h. After dialysis, the obtained residues were freeze-dried for further analysis of macronutrients content. The in vitro digestion of breads was done in 8 replicates (two samples from each bread).

### Statistical analysis

The results are presented as mean ± standard deviations. For multiple comparisons, one-way analysis of variance (ANOVA) was used. Differences among means were tested for significance using Duncan’s multiple range test (MRT). Significance was set at *p* ≤ 0.05. The statistical analysis was performed using the statistical software Statistica 8.0 (StatSoft Inc., USA).

## Results and discussion

### Proximate composition of the wholemeal flour (WRF) and HFRF

As expected, the WRF and the HFRF differed significantly in contents of major macronutrients (Table 1). Compared to the WRF, the HFRF had a significantly higher content of minerals, fat, protein, TDF and its major components, but significantly less of total starch. More specifically, the HFRF contained more than twice of the TDF and almost three times more of the SDF than the WRF. Generally, the SDF is considered as an important component of the human diet. Most health benefits associated with intake of cereal-based products are attributed to the SDF fraction, which originates mainly from the outer rye kernel layers (Slavin 2003; Ye et al. 2012). In this study, the concentrations of major fibre components, namely arabinoxylan, β-glucan and fructan in the HFRF were significantly higher (*p* ≤ 0.05) than those in the WRF by 2.0, 1.8 and 1.6 folds, respectively. The content of TDF and its components was correlated inversely with the content of total starch. The ratio of TDF to total starch in the HFRF was about six times higher than in the WRF.

**Table 1:** Chemical composition of the wholemeal rye flour (WRF) and the high-fibre rye flour (HFRF), expressed as % of d.m.

Components	Wholemeal rye flour (WRF)	High-fibre rye flour (HFRF)	*p* value
Minerals (ash)	2.0 ± 0.0	4.5 ± 0.1	4.51E−07
Lipids	2.1 ± 0.1	3.0 ± 0.3	0.0052
Protein (nitrogen × 6.25)	12.3 ± 0.2	18.5 ± 0.2	1.87E−06
Total starch	58.1 ± 0.3	24.5 ± 0.2	4.83E−09
Total reducing sugars	1.8 ± 0.1	2.9 ± 0.2	0.0004
Total dietary fibre (TDF)	17.7 ± 0.3	42.1 ± 0.3	9.42E−08
Soluble (SDF)	2.9 ± 0.1	8.1 ± 0.3	5.00E−06
Insoluble (IDF)	14.8 ± 0.3	34.0 ± 0.2	5.58E−08
Total arabinoxylan	8.9 ± 0.2	18.3 ± 0.5	5.52E−06
Water-soluble (WE)	2.6 ± 0.1	4.8 ± 0.3	0.0001
Water-insoluble (WUE)	6.3 ± 0.3	13.5 ± 0.2	3.11E−06
Total β-glucan	2.3 ± 0.1	4.1 ± 0.2	0.0002
Water-soluble (WE)	1.1 ± 0.1	1.3 ± 0.1	0.3486
Water-insoluble (WUE)	1.2 ± 0.1	2.8 ± 0.1	3.82E−06
Fructan	4.2 ± 0.1	6.9 ± 0.2	2.21E−05

*d.m.* dry matter

Values are mean ± standard deviation

Moisture contents of WRF and HFRF were 13.7% and 10.7%, respectively

### Effect of HFRF enrichment level on physicochemical characteristics of wholemeal rye bread

As shown in Table 2, no significant differences between CB and supplemented breads were observed in the loaves volume and the specific volume of bread. The total titratable acidity values of breads were within the normal range for rye sourdough bread (Boskov Hansen et al. 2002). Compared to the CB, the total colour difference (∆E*) and the browning index values (BI) of crusts and crumbs gradually increased with the HFRF supplementation level. In both crust and crumb, the colour changed from brown to dark brown, but crusts colour changed more than the crumbs. The crust colour change from brown to dark brown was expected because the HFRF contained more reducing sugars than the WRF (Table 1). It is possible that during the HFRF preparation some starch granules were damaged mechanically leading to the formation of maltooligosaccharides with reducing properties. It has been reported that high level of reducing sugars in bread flour was associated with the formation of dark Maillard and caramelization products during bread baking (Peressini and Sensidoni 2009; Koletta et al. 2014).

**Table 2:** Effect of addition high-fibre rye flour on the physicochemical properties and sensory evaluation of control and supplemented breads

	Control bread	Supplemented breads		
	CB	SB30	SB40	SB50
*Loaf volume and acidity*				
Loaf volume (cm^3^/100 g of flour)	275^a^ ± 13	261^a^ ± 16	264^a^ ± 14	258^a^ ± 18
Specific volume (cm^3^/g of bread)	1.71^a^ ± 0.03	1.65^a^ ± 0.04	1.64^a^ ± 0.04	1.59^a^ ± 0.05
Total titratable acidity (°)	8.9^a^ ± 0.2	9.3^b^ ± 0.1	9.4^b^ ± 0	9.3^b^ ± 0.2
*Colour (CIELab)*				
∆E*				
Crust	–	3.1^a^ ± 0.6	3.8^a^ ± 0.7	7.1^b^ ± 0.4
Crumb	–	1.0^a^ ± 0.3	1.3^a^ ± 0.1	2.9^b^ ± 0.3
BI				
Crust	66.8^a^ ± 0.3	69.9^b^ ± 0.4	70.5^b^ ± 0.2	73.6^c^ ± 0.3
Crumb	55.3^a^ ± 0.1	56.1^b^ ± 0.3	56.5^b^ ± 0.2	57.7^c^ ± 0.2
*Sensory analysis and acidity of breads*				
Shape, volume, exterior appearance (max. 2 points)	2	2	2	1
Crust (max. 2 points)	2	2	2	1
Crumb (max. 10 points)	9	8	8	6
Porosity (max. 5 points)	4	4	3	3
Elasticity (max. 3 points)	3	3	3	2
Structure (max. 2 points)	2	1	1	1
Taste and smell (max. 5 points)	5	5	5	4
Acidity (max. 1 points)	1	1	1	1
Total points (max. 20 points)	19	18	17	13

Values are mean ± standard deviation

∆E*-total colour difference

*BI* browning index

Values within rows followed by the different letters are significantly different (*p* ≤ 0.05)

### Sensory evaluation

The sensory evaluation (Table 2) showed that the HFRF supplementation had a slight negative effect on both external and internal characteristics of the supplemented breads. The shape of all breads except SB50 was standard, and the crust was plain without cracks. Compared to the CB, crumb properties of the supplemented breads differed slightly. More specifically, with a higher level of HFRF supplementation, the crumb structure was slightly tighter and. the crumb of the SB50 was somewhat *sticky. The a*roma, smell, and taste of SB30 and SB40 were scored as 5 points (maximum score) and SB50 as 4 points. The SB50 had a slightly bitter taste and a gentle aroma of rye bran. The panellists classified the SB30 and SB40 as acceptable with average overall acceptability scores of 18 and 17, respectively. Consequently, both SB30 and SB40 were classified as breads of good quality, whereas the SB50 with the overall average score of 13 was classified as a bread of unsatisfactory quality (Drews and Seibel 1976).

Several studies showed that replacement of wheat flour with dietary fibre from various sources usually produces bread with lower loaf volume, reduced porosity and elasticity of crumb, and darker crust and crumb (Symonds and Brennan 2004; Peressini and Sensidoni 2009; Angioloni and Collar 2011; Rizzello et al. 2012; Almeida et al. 2013; Koletta et al. 2014). In addition, it has been documented that supplemented wheat bread might lack consumer acceptance. There is a consensus that a dilution of gluten causes these undesirable effects of fibre supplementation in wheat flour blends used for baking, modification of starch-gluten matrix and/or effect of fibre on starch pasting properties (Symonds and Brennan 2004; Peressini and Sensidoni 2009; Ragaee et al. 2011). All these studies refer to wheat bread and research on the sensory quality of rye bread is limited. The effect of partial replacement of wholemeal rye flour with the high-fibre cereal products on sensory evaluation of supplemented rye bread has not, to our knowledge, been discussed in literature.

In the case of wholemeal rye flour, the content of arabinoxylan, and especially its highly viscous soluble fraction is considered a significant factor affecting rye bread loaf volume and physical properties of crumb (Poutanen et al. 2014). It is possible that the soluble arabinoxylan in the rye flour blends used in this study partly mitigated the undesirable effects of starch dilution on bread volume and crumb texture. Soluble fibre might add viscosity to the food matrix during digestion, slowing digestion and nutrient absorption and hence reducing the glycemic index (GI).

Many consumers find wholemeal wheat bread to be unattractive and unpalatable, due to the colour, flavour (bitterness) and texture (grittiness). However, it may be possible to achieve a significant increase in the content of fibre and other beneficial components by increasing the flour extraction rate by a limited amount with minimal effects on acceptability. This study showed that replacement the WRF with 30 and 40% HFRF allows producing breads with acceptable external properties and sensory characteristics.

### Chemical composition and nutrient and energy content of bread

Chemical composition and nutritional data are in Table 3. All tested breads were a good source of minerals measured as ash. The supplemented breads contained approximately 20% more minerals than the CB. The amounts of lipids and protein in all breads were similar. Compared to CB, all supplemented breads had significantly lower content of available saccharides, defined as the sum of available starches (RDS and SDS) and free reducing sugars, and a significantly higher TDF content. The amounts of total starch and its RDS fraction in the supplemented breads were significantly lower than in the CB. The RS content was similar to that in a recent study in wheat bread enriched with wholemeal rye flour (Koletta et al. 2014), but it was lower than the content of RS in wheat bread fortified with whole grain rye flour reported by Ragaee et al. (2011) and Tas and El (2000).

**Table 3:** Nutrients, energy, and in vitro products content of control and supplemented breads with 30% (SB30), 40% (SB40) and 50% (SB50) of HFRF as eaten

Component (g/100 g)^1^	Control bread CB	Supplemented bread		
		SB30	SB40	SB50
Water	44.6^a^ ± 0.1	44.9^b^ ± 0.1	45.0^b^ ± 0.1	45.4^c^ ± 0.1
Minerals (ash)	1.8^a^ ± 0.0	2.1^b^ ± 0.2	2.2^b^ ± 0.1	2.4^b^ ± 0.1
Lipids	1.0^a^ ± 0.1	1.2^a^ ± 0.1	1.2^a^ ± 0.2	1.3^a^ ± 0.0
Protein (Nitrogen × 6.25)	7.1^a^ ± 0.2	7.7^b^ ± 0.1	7.9^b^ ± 0.1	8.2^c^ ± 0.1
Total saccharides	32.3^d^ ± 0.3	27.3^c^ ± 0.3	25.7^b^ ± 0.4	23.9^a^ ± 0.6
Available saccharides	31.7^d^ ± 0.9	27.9^c^ ± 0.5	25.3^b^ ± 0.5	23.4^a^ ± 0.7
Total starch	30.6^d^ ± 0.8	25.3^c^ ± 0.5	23.4^b^ ± 0.3	21.4^a^ ± 0.5
Rapidly digestible starch (RDS)	28.0^d^ ± 1.2	23.3^c^ ± 0.3	21.7^b^ ± 0.3	19.7^a^ ± 0.8
Slowly digestible starch (SDS)	2.0^a^ ± 0.5	1.5^a^ ± 0.2	1.3^a^ ± 0.3	1.2^a^ ± 0.2
Resistance starch (RS)	0.6^a^ ± 0.2	0.4^a^ ± 0.1	0.4^a^ ± 0.2	0.5^a^ ± 0.1
Total free reducing sugars	1.7^a^ ± 0.2	2.0^b^ ± 0.1	2.3^c^ ± 0.1	2.5^c^ ± 0.2
Free glucose	0.3^a^ ± 0.0	0.7^b^ ± 0.1	0.8^b^ ± 0.2	0.7^b^ ± 0.2
Rapidly available glucose (RAG)	31.4^c^ ± 1.2	26.6^b^ ± 0.5	24.9^a^ ± 0.4	22.6^a^ ± 0.9
Starch Digestion Index (SDI) (%)	91.5^a^ ± 1.4	91.9^a^ ± 0.9	92.7^a^ ± 0.9	92.1^a^ ± 1.1
Total dietary fibre (TDF)	8.9^a^ ± 0.3	12.1^b^ ± 0.4	13.1^c^ ± 0.1	14.8^d^ ± 0.5
Soluble (SDF)	2.1^a^ ± 0.2	2.7^b^ ± 0.1	3.1^c^ ± 0.1	3.3^c^ ± 0.3
Insoluble (IDF)	6.8^a^ ± 0.3	9.4^b^ ± 0.5	10.0^c^ ± 0.3	11.5^d^ ± 0.6
Total arabinoxylan	4.9^a^ ± 0.2	6.3^b^ ± 0.1	6.9^c^ ± 0.1	7.5^d^ ± 0.3
Water-soluble (WE)	1.8^a^ ± 0.1	2.2^b^ ± 0.2	2.2^b^ ± 0.1	2.3^b^ ± 0.2
Water-insoluble (WUE)	3.1^a^ ± 0.2	4.2^b^ ± 0.1	4.7^c^ ± 0.1	5.2^d^ ± 0.2
Total β-glucan	1.2^a^ ± 0.1	1.4^b^ ± 0.1	1.5^b^ ± 0.1	1.5^b^ ± 0.2
Water-soluble (WE)	0.6^a^ ± 0.1	0.7^a^ ± 0.0	0.6^a^ ± 0.1	0.7^a^ ± 0.1
Water-insoluble (WUE)	0.6^a^ ± 0.1	0.7^a^ ± 0.2	0.9^a^ ± 0.1	0.8^a^ ± 0.1
Fructan	1.4^a^ ± 0.0	1.5^a^ ± 0.1	1.5^a^ ± 0.1	1.7^a^ ± 0.1
Energy				
kcal/100 g	185^b^ ± 3	180^b^ ± 1	173^a^ ± 1	171^a^ ± 2
kJ/100 g	789^b^ ± 13	767^b^ ± 7	734^a^ ± 4	726^a^ ± 8

Values are mean ± standard deviation

^1^Except where noted

RDS—starch hydrolysed within 20 min of incubation

SDS—starch digested between 20 and 120 min of incubation

RS—starch not hydrolysed within 120 min of incubation

RS was calculated as difference between total starch content and sum of RDS and SDS

Values within rows followed by the different letters are significantly different (*p* ≤ 0.05)

The RAG contents and the SDI values of all breads are in Table 3. Both parameters are used to monitor and control carbohydrates intake, glucose and insulin balance in people with diabetes (Englyst et al. 1996). The supplemented breads had significantly lower RAG content than the CB, but the SDI values of all breads were similar. Ingestion of bread with a high RAG content might cause a rapid increase of plasma glucose and insulin levels, which have been associated with health complications such as diabetes, cardiovascular disease, and obesity (Englyst et al. 1996; Lappi et al. 2014). Englyst et al. (1996) analysed the nutritionally important starch fractions content and glycemic index (GI) of 39 commonly used starchy foods. They reported a highly significant positive correlation between the GI and both RDS and RAG. Since the RAG value relates to the food as eaten and includes both RDS and free glucose, it was proposed as a better indicator for blood glucose and insulin response than the SDI (Englyst et al. 1996). The SDI values of breads in this study were slightly lower than those white rye bread and corn bread reported by Tas and El (2000). Ragaee et al. (2011) found strong correlations between starch digestibility (i.e., contents of RDS and SDS) and contents of both IDF and SDF in wheat breads enriched in fibre.

Including the fructan, the TDF contents in the supplemented breads as eaten were higher from 32 to 60% for the SB30 and the SB50, respectively compared to the CB. Moreover, the supplemented breads had significantly more SDF, but the SDF contents of the TDF in all breads remained stable on average 35%. It means that approximately three standard weighing 56 g slices of all tested supplemented breads could provide recommended daily intake for TDF (25 g/day) not necessarily at one eating occasion (EFSA 2010). The supplemented breads contained about 50% more TDF including fructan than various types of Swedish commercial wholemeal rye soft breads enriched in rye brans analysed recently by Rakha et al. (2010).

Compared to the CB, the supplemented breads had significantly higher contents of arabinoxylan and its WE and WUE fractions as well as β-glucan. In contrast, the WE β-glucan and WUE β-glucan as well as fructan contents in the supplemented breads were similar when compared to the CB. The content of fructan in all breads decreased significantly during bread making by about 50%. These results are in agreement with Boskov-Hansen et al. (2002) who observed a 45% loss of fructan during wholemeal rye bread making. They hypothesized that the loss was a consequence of fructan breakdown to lower molecular weight sugars and partial fermentation by yeast or/and other sourdough microflora. Several human studies found positive effects of fructan, especially short chain fructooligasaccharides on glucose and lipid metabolism as well as their prebiotic and bifidogenic properties (Zhang and Hamaker 2010; Maziarz 2013). Thus, the relatively high fructan content in the supplemented breads (2.5–2.9 g/3 slices) might have a beneficial effect on diet particularly that the estimated daily average fructans intake from natural sources in Western countries is lower than 3.5 g, whereas the amount needed for beneficial effects is 15 g/day (Maziarz 2013).

All supplemented breads had 10–20% lower energy content than the CB. They also contained about 10–20% less energy when compared to experimental high-fibre wheat breads (Angioloni and Collar 2011). Although all tested supplemented breads had improved nutritional characteristics compared to the CB, the CB50 bread had unacceptable sensory characteristics and SB40 had a better nutritional quality than SB30. Therefore, in the further in vitro digestibility experiments only the SB40 and the CB were included for comparison.

### In vitro enzymatic digestion

An in vitro model was use to simulate bread digestion in the upper gastrointestinal tract. The chemical composition of CB and SB40 after in vitro enzymatic digestion are in Table 4. During the enzymatic treatment all components of both breads were partly hydrolysed to a different extent. The overall digestibility was lower for the SB40 than CB (65 and 73%, respectively). In both breads approximately 95% of starch and 75% of protein were digested. The DF and its fractions were highly resistant to digestive enzymes. In the SB40 and the CB, only 9% and 11% of the TDF were irrecoverable and slightly more of the SDF (14–16%) than the IDF (8–9%) were lost. It is feasible that small losses of the DF fractions were due to their slight depolymerisation during digestion. In the enzymatic digestion of both breads, almost similar amounts of arabinoxylan and β-glucan (27–32% and 37–38%, respectively) were lost. Compared to WE, WUE fractions of arabinoxylan and β-glucan were lost to a greater extent, because after in vitro digestion soluble DF components were partially separated by dialysis from insoluble residues. Moreover, the majority of fructans (78–80%) as soluble compounds of DF was eliminated.

**Table 4:** Chemical composition of control and supplemented breads after the in vitro digestion (expressed as % d.m.) and undigested residues of dry weight and other components (expressed as % of original)

Component	Control bread (CB)		Supplemented bread (SB40)	
	After digestion	Residue (% original substrate)	After digestion	Residue (% original substrate)
Dry weight	27.2^a^ ± 0.2	27	35.2^b^ ± 0.5	35
Protein (Nitrogen × 6.25)	10.9^a^ ± 0.2	23	11.0^a^ ± 0.2	27
Total starch	13.0^b^ ± 0.6	6	6.5^a^ ± 0.2	5
Total dietary fibre (TDF)	52.8^a^ ± 0.6	89	61.3^b^ ± 1.2	91
Soluble (SDF)	11.7^a^ ± 0.1	84	13.7^b^ ± 0.2	86
Insoluble (IDF)	41.1^a^ ± 0.6	91	47.6^b^ ± 1.0	92
Total arabinoxylan	22.1^a^ ± 0.9	68	26.1^b^ ± 0.9	73
Water-soluble (WE)	8.5^b^ ± 0.3	71	7.5^a^ ± 0.2	66
Water-insoluble (WUE)	13.6^a^ ± 0.6	66	18.6^b^ ± 1.0	77
Total β-glucan	5.0^a^ ± 0.1	63	4.8^a^ ± 0.2	62
Water-soluble (WE)	2.1^b^ ± 0.1	53	1.8^a^ ± 0.2	58
Water-insoluble (WUE)	2.9^a^ ± 0.1	73	3.0^a^ ± 0.2	65
Fructan	1.9^a^ ± 0.1	20	1.7^a^ ± 0.1	22

Values are mean ± standard deviation

*d.m.* dry matter

The residue dry weight, protein, starch, dietary fibre and its components after digestion as a percentage of those originally present in the breads

Values within rows followed by the different letters are significantly different (*p* ≤ 0.05)

Our results for the composition of breads’ substrates after in vitro digestion were difficult to compare with existing data since only a few studies on rye or wheat breads have been reported. Most research examined in vitro digestibility of white wheat bread and was carried out using different experimental models than in this study (Symonds and Brennan 2004; Angioloni and Collar 2011; Rizzello et al. 2012; Nordlund et al. 2013). However, the results from this study were in the good agreement with results reported by Aura et al. (1999), who found that overall, starch and protein digestibility for wholemeal rye bread were 78%, 91% and 75%, respectively. They also found the slight losses of TDF and arabinoxylan and β-glucan from the wholemeal rye bread after in vitro digestion. Karppinen et al. (2000) and Nordlund et al. (2012) using the same in vitro procedure as in our study, demonstrated that the extent of DF components losses during digestion of high-fibre cereal-based products such as brans or milling by-products appeared to be dependent on the source and nature of the fibre. In turn, in a small in vivo study conducted in seven women, Viskar et al. (1996) concluded that the digestibility of protein, non-starch polysaccharides, and energy content were significantly lower for the diets containing the wholemeal rye breads compared with the low-fibre control diets.

**Fig. 2:**
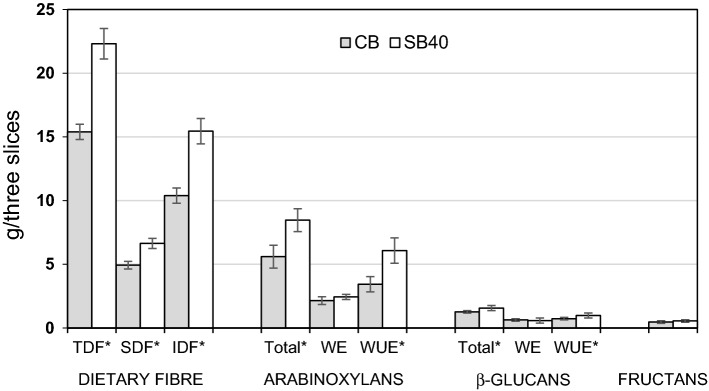
The amounts of dietary fibre and its components from supplemented (SB40) and control bread (CB) after in vitro digestion. Data are mean ± standard deviation; the stars next to fibre component denote significant differences between means for SB40 and CB at *p* ≤ 0.05

Based on the in vitro small intestine digestion model, we compared the amounts of fibre components residues from both breads that in vivo would most likely reach the colon (Fig. 2).

The values were calculated for three standard slices of the CB and SB40 as eaten. In the human digestive track these indigestible carbohydrates would be partly fermented in the colon by intestinal microflora into short-chain fatty acids such as acetate, butyrate, and propionate, which have been associated with lowered intestinal pH and reduced risk of several cancers in humans (Zhang and Hamaker 2010). In this study, we found that undigested residue from three slices of the SB40 contained significantly more TDF, SDF, IDF, total arabinoxylan and total β-glucan and their WUE fractions than the CB. However, the amounts of the WE fractions of both arabinoxylan and β-glucan and fructan for both breads were similar.

### Limitations

This study has some limitations. First, supplementation of bread in the HFRF was limited to three levels. However, the study goal was to identify the highest level of supplementation that would both, improve nutritional quality and have acceptable physicochemical and sensory properties. Further studies should identify an optimal level of supplementation of bread in the HFRF. Second, the study used only a traditional bread making technology. Other technologies that would potentially improve quality of bread such as the use of enzymes or additives and/or manipulate sourdough fermentation, proofing time and temperatures should be tested in future studies. Third, the study used only in vitro enzymatic digestion as a model and the obtained substrates should be subjected to further fermentation studies using in vitro colon model. Hence, any in vivo study especially a randomized clinical trial would be premature at this point.

## Conclusion

The dietary fibre content of wholemeal rye flour used in bread making could be increased by supplementing with high-fibre rye flour (HFRF) separated during milling. Among breads baked using flour supplemented with HFRF (30%, 40%, 50%), breads with 30 and 40% of HFRF (SB30 and SB40) had sensory and physicochemical characteristics similar to commonly consumed rye bread (CB). Compared to the CB, the bread with 40% of HFRF had significantly higher content of protein, soluble and insoluble fractions of dietary fibre, arabinoxylan, and β-glucan, but lower content of available saccharides, including rapidly available glucose, and energy. An in vitro overall digestibility of the SB40 was lower than that of the CB. An undigested residue from SB40 contained significantly more TDF, SDF, IDF, total arabinoxylan and total β-glucan and their water insoluble (WUE) but not water soluble (WE) fraction than the CB. The SB40 could be considered as an option to commonly consumed wholemeal rye bread.
